# Broadband imaging with one planar diffractive lens

**DOI:** 10.1038/s41598-018-21169-4

**Published:** 2018-02-12

**Authors:** Nabil Mohammad, Monjurul Meem, Bing Shen, Peng Wang, Rajesh Menon

**Affiliations:** 1 Department of Electrical and Computer Engineering, University of Utah, Salt Lake City, UT 84112 USA; 2 MACOM Technology Solutions, Ithaca, NY 14850 USA; 3 Department of Medical Engineering, California Institute of Technology, Pasadena, CA 91125 USA

## Abstract

We demonstrate imaging over the visible band using a single planar diffractive lens. This is enabled via multi-level diffractive optics that is designed to focus over a broad wavelength range, which we refer to as an achromatic diffractive lens (ADL). We designed, fabricated and characterized two ADLs with numerical apertures of 0.05 and 0.18. Diffraction-limited focusing is demonstrated for the NA = 0.05 lens with measured focusing efficiency of over 40% across the entire visible spectrum (450 nm to 750 nm). We characterized the lenses with a monochromatic and a color CMOS sensor, and demonstrated video imaging under natural sunlight and other broadband illumination conditions. We use rigorous electromagnetic simulations to emphasize that ADLs can achieve high NA (0.9) and large operating bandwidth (300 nm in the visible spectrum), a combination of metrics that have so far eluded other flat-lens technologies such as metalenses. These planar diffractive lenses can be cost-effectively manufactured over large areas and thereby, can enable the wide adoption of flat, low-cost lenses for a variety of imaging applications.

## Introduction

Refractive lenses are bulky and often challenging to incorporate into imaging systems that have restricted form factors. Diffractive optics, on the other hand, may be planar and lightweight^1^. However, conventional diffractive optics are not typically used for imaging because of significant off-axis and chromatic aberrations^2,3^ as well as low broadband focusing efficiencies. Recently, planar metalenses have been used for imaging^4,5^. Unfortunately, metalenses require subwavelength features and large aspect ratios, making them impractical for low-cost manufacturing over large areas. In addition, they usually suffer from polarization sensitivity^6–8^ and possess significant chromatic aberrations^9–13^. Metalenses with conical wavefronts have been demonstrated for focusing^14,15^. However, these suffer from relatively low transmission efficiency, require very small features (~100 nm) and therefore are difficult to scale to larger apertures. Here, we demonstrate that metalenses are not required for imaging light intensities, a scalar property of the electromagnetic field. Diffractive optics with super-wavelength features and relatively low aspect ratios, which are far simpler to fabricate, are sufficient for imaging light intensities. However, we note that metasurfaces are required to manipulate vector properties of light such as polarization^16^. We further emphasize that we previously demonstrated water-immersion diffractive lenses with numerical aperture (NA) as high as 1.43^17^.

Previously, we utilized the concept of broadband diffractive optics to design broadband holograms^18^ and to demonstrate broadband spectrum splitting and concentration^19,20^, phase masks for 3D lithography^21^ and cylindrical lenses with super-achromatic performance over the entire visible band^22^. Here, we design, fabricate and characterize broadband diffractive optics as planar lenses for imaging. Broadband operation is achieved by optimizing the phase transmission function for each wavelength carefully to achieve the desired intensity distribution at that wavelength in the focal plane. We designed these achromatic diffractive lenses (ADLs) by maximizing the focusing efficiency at the design wavelengths ranging from 450 nm to 750 nm in steps of 50 nm. We numerically investigated multiple sampling schemes in wavelength and decided that our chosen scheme provided a good compromise between computational cost and average focusing efficiency. Each lens is comprised of concentric circular rings of width = 3 μm and the height of each ring is varied between 0 and a maximum value (which was 2.4 μm and 2.6 μm for the NA = 0.05 and 0.18 lenses, respectively). We designed and experimentally characterized two lenses each of focal length, f = 1 mm and numerical aperture (NA) of 0.05 and 0.18, respectively. All design parameters are summarized in the Supplementary information (Table S1). Recently, broadband diffractive lenses have been applied to imaging^23^. However, due to very low resolution of these lenses, significant image blurring is observed, which requires significant post-processing to obtain sharp images. In contrast, our approach is able to maintain the quality of the images comparable to that achievable with more complex systems of lenses.

**Figure 1:**
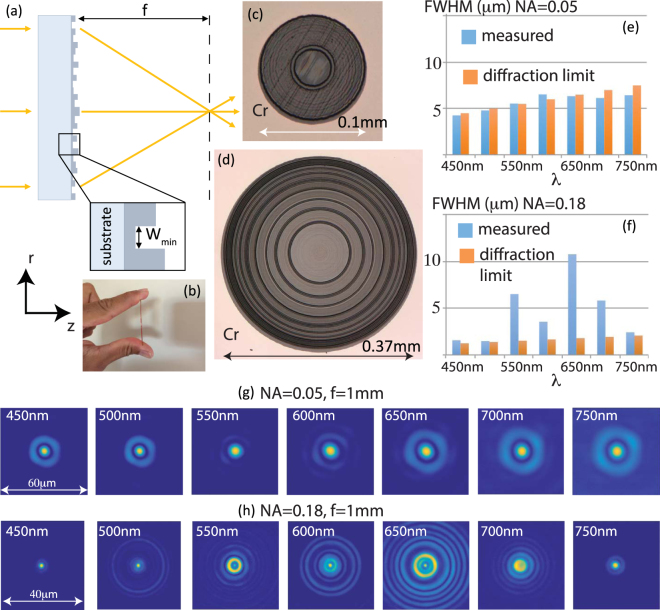
(**a**) Schematic of a flat-lens design. The structure is comprised of concentric rings of width, W_min_ and varying heights. (**b**) Photograph of one fabricated lens. Optical micrographs of (**c**) NA = 0.05 and (**d**) NA = 0.18 lenses. Focal length is 1 mm. Measured full-width at half-maximum (FWHM) of the focal spot as a function of wavelength for (**e**) NA = 0.05 and (**f**) NA = 0.18 lenses. Measured focal spots as a function of wavelength for (**g**) NA = 0.05 and (**h**) NA = 0.18 lenses.

The diffractive lens can be accurately modeled by scalar diffraction theory in the regime of Fresnel approximation^24,25^. We utilized a modified version of direct-binary search to optimize the height profile of each lens. Details of the design process have been described elsewhere^22^. The on-axis focusing efficiency averaged over all the design wavelengths is used as the metric for optimization. Grayscale lithography was used to fabricate the lenses in a positive photoresist (Shipley 1813) spin coated on a glass wafer^22^. A photograph of one of the lenses is shown in Fig. 1(b) to confirm that this diffractive lens is indeed flat. Optical micrographs of the fabricated lenses are shown in Fig. 1(c,d), for NA = 0.05 and 0.18, respectively. Figure 1(e,f) shows the measured full-width at half-maximum (FWHM) of the focal spot as a function of wavelength for NA = 0.05 and 0.18 lenses, respectively. In Fig. 1(e), it is clear that the fabricated device is able to achieve diffraction-limited performance to within 5% of the diffraction-limited value. The NA = 0.18 lens fails to achieve diffraction-limited spot size for some of the design wavelengths. Nevertheless, we show below that both lenses are capable of forming reasonably good quality images. In the case of the NA = 0.18 lens, simple image processing such as deconvolution can significantly improve the imaging performance as well. The corresponding simulated focal spots (see Supplementary Figure S1) indicate that there is good agreement between experiments and simulations for both lenses. We note that the streaks that are visible in Fig. 1(c) arise from a stage calibration error in our patterning tool, which we believe has some effect on the experimental focusing efficiency as described later. These streaks can be eliminated with proper stage calibration. The diffraction rings seen in the focal spots of the NA = 0.18 lens may be mitigated by the use of smaller ring widths and careful choice of the metric for optimization.

A collimated beam from a super-continuum source equipped with a tunable filter illuminates the lens (see supplementary information)^22^. The light distribution at the focus for each lens was then magnified by an objective-tube lens system and recorded on a monochrome sensor (see supplementary information for details) and are shown in Fig. 1(g,h) for NA = 0.05 and 0.18 lenses, respectively.

**Figure 2:**
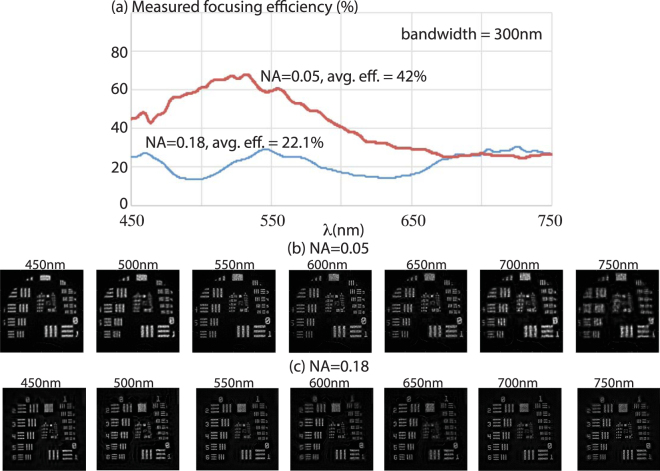
(**a**) Measured focusing efficiency as a function of wavelength for the 2 lenses shown in Fig. 1. Images captured on a monochrome sensor of the Air Force resolution target in transmission at various illumination wavelengths for (**b**) NA = 0.05 and (**c**) NA = 0.18 lenses. Details of the experiments are in the text and in the supplementary information. Blind deconvolution was applied to these images.

The focusing efficiency, defined as the ratio of the integrated power over a circular aperture with diameter 18 μm in the focal plane to the total power over the lens aperture as a function wavelength was also measured (Fig. 2a). Measurement details are included in the supplementary information. The simulated focusing efficiencies are included in the supplementary information (Fig. S2). Numerical analysis of the fabrication errors included in the supplementary information (see Fig. S3) seems to suggest that fabrication errors are one possible reason for the reduction in the measured focusing efficiencies. Nevertheless, the measured efficiency averaged over the 300 nm bandwidth of 42% and 22.1% for NA = 0.05 and 0.18 lenses, respectively, is considerably larger than the averaged efficiency of a comparable metalens over a much smaller bandwidth (60 nm)^26^. We also point out that the focusing efficiencies can be increased by allowing for smaller zone widths as discussed for the case of high-NA lenses later. We further note generally it is more challenging to design a lens with a larger aperture while maintaining the imaging performance. This might be mitigated in the future by combining refractive and diffractive power into a single aperture.

To demonstrate imaging capability, we used a standard test chart (USAF 1951) with a diffuser behind it as the object and imaged it onto the same monochrome sensor using each flat lens. Details of the experiment are described in the supplementary information. We used the same illumination source (varying center wavelength and bandwidth = 10 nm) as was used for focal spot characterization. A simple blind deconvolution method was applied to the raw images and the results are presented in Fig. 2(b,c) for NA = 0.05 and 0.18 lenses, respectively. It is clear that our lenses are able to form relatively good images at all the wavelengths.

**Figure 3:**
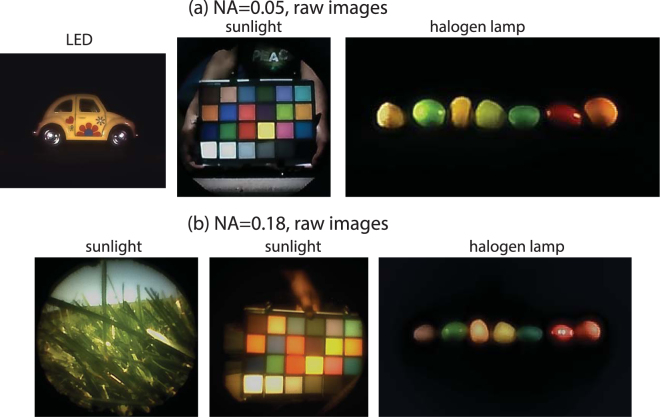
Example photographs taken with a camera consisting of only a single flat lens and a conventional color CMOS sensor. An IR-cut filter is placed in front of the lens for images taken under sunlight. Video data taken with these cameras are included as supplementary material.

Finally, we built a color camera by placing the flat lens with a conventional CMOS sensor (DFM 72BUC02-ML, The Imaging Source). A variety of test images were captured using both artificial lighting as well under ambient sunlight. In the latter case, a conventional IR-cut filter was placed in front of the lens. The results are summarized in Fig. 3. Since our sensor can capture video data, we have also included two examples of videos as supplementary information for each of the two lenses.

As we have pointed out before, these diffractive lenses are also polarization-insensitive^22^. This ensures that the lenses perform equally well for any polarization input, which is important for general imaging applications. All experiments reported here were conducted with randomly polarized incident light.

In a diffractive lens, the maximum achievable NA is determined by λ/(2*w), where w is the minimum feature size (constrained by fabrication). In order to achieve NA comparable to that of a mobile-phone camera (NA~0.3), one needs w ~0.83 μm. We can expect the maximum pixel height to be similar to what we have used here. This means that for many photography applications, a diffractive lens would need an aspect ratio of only 1.7. This is considerably simpler to fabricate than a visible-wavelength metalens, where feature widths of <50 nm and aspect ratios >15 are required^5^.

**Figure 4:**
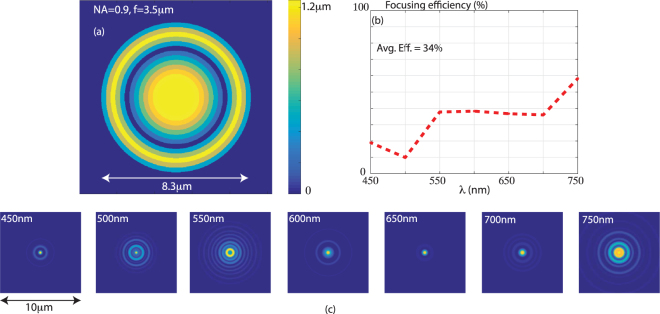
NA = 0.9 diffractive lens. (**a**) Design details. (**b**) Simulated focusing efficiency as a function of wavelength. (**c**) Simulated focal spots at various design wavelengths in the visible spectrum.

For ease of fabrication, we chose to demonstrate lenses with relatively low NA (large F/#). It is possible to overcome this limitation with improved fabrication processes. To illustrate this point, we designed a lens with NA = 0.9 and f = 3.5 μm using minimum zone width of 0.25 μm and maximum zone height of 1.25 μm. Note that the maximum aspect ratio for this lens is 5:1 and the minimum feature is considerably larger than those required in metalenses. For ease of computation, we applied the same scalar diffraction model as before during the design process. The basic design methodology including the figure of merit was the same as for the low-NA lenses. Finally, we applied rigorous electromagnetic modeling via the finite-difference time-domain (FDTD) method to analyze the performance of the optimized design, which is shown in Fig. 4(a). The simulated focusing efficiency defined as the ratio of power inside 3 times full-width at half-maximum to that inside the lens aperture is plotted as a function of wavelength in Fig. 4(b), and the calculated average efficiency is 34% across an operating bandwidth of 300 nm. The simulated focal spots at the design wavelengths are shown in Fig. 4(c). Although this preliminary design does not achieve diffraction-limited focusing at all the design wavelengths, these are sufficient for good image formation via post-processing using a-priori information of the focused spots as illustrated by the images formed by the fabricated NA = 0.18 lens above^23^. Thereby, we confirm that our multilevel diffractive lens can achieve high-NA and large operating bandwidth, a combination of requirements that have so far eluded single-element imaging systems.

We show here that planar diffractive lenses, when designed properly and fabricated carefully are sufficient for broadband imaging. By extending the fabrication process to industry-relevant lithographic scales and large area replication via nanoimprinting^27^, it is possible to envision planar lenses enabling imaging with very thin form factors, low weights and low costs. Therefore, we believe that our approach will lead to considerably simpler, thinner and cheaper imaging systems.

## Methods

The achromatic lenses were patterned on a photoresist film atop a glass wafer using grayscale laser patterning using a Heidelberg Instruments MicroPG101 tool. The exposure dose was varied as a function of position in order to achieve the multiple height levels dictated by the design.

The devices were characterized on an optical bench by illuminating them with broadband collimated light, whose spectral bandwidth could be controlled by a tunable filter. The focus of the lenses were captured on a monochrome CMOS sensor for characterization of the PSF. Imaging performance of the lenses were tested in prototype cameras as described in the main text with various color objects under various illuminations including ambient sunlight.

## Electronic supplementary material

Video using NA=0.05 flat lens

Video using NA=0.18 flat lens

Supplementary Information
