# Supplementary material for: Broadband imaging with one planar diffractive lens
Source: Sci Rep. 2018 Feb 12;8:2799. doi: 10.1038/s41598-018-21169-4 (PMC5809505; doi:10.1038/s41598-018-21169-4)
Supplement: Supplementary file 3 — Supplementary Information [file 41598_2018_21169_MOESM3_ESM.pdf]

Supplementary Information

Broadband imaging with one planar diffractive lens

Nabil Mohammad,[1] Monjurul Meem,[1] Bing Shen,[2] Peng Wang,[3] and Rajesh Menon[1,*]

[1] Department of Electrical and Computer Engineering, University of Utah, Salt Lake City UT 84112
[2] MACOM Technology Solutions, NY 14850
[3] Department of Medical Engineering, California Institute of Technology, Pasadena CA 91125
* Correspondence to rmenon@eng.utah.edu

1. Design methodology

Our broadband lens is comprised of concentric rings of varying heights as illustrated in Fig. 1(a). The widths of the rings may be varying as well. In this manuscript, the widths of the rings were kept the same. The diameter of the lens is determined by the numerical aperture of the design, focal length and the longest wavelength of operation. The field in the focal plane is computed using scalar diffraction theory at each wavelength and the corresponding focusing efficiency is also computed. The focusing efficiency is defined as the power focused within 3 X FWHM divided by the total incident power at a given wavelength. The goal of our optimization based design is to maximize the focusing efficiency averaged over all the wavelengths of interest by varying the heights of the rings comprising the lens. We refer to this as the figure of merit (FOM). We utilized the modified binary-search algorithm to perform this optimization (see flowchart below). At first, an initial guess of height distribution is generated (usually a random distribution). In one iteration, all ring-heights are perturbed in a pre-designed manner (a random sequence). A positive unit perturbation ($+\Delta h$) is tried. If the updated FOM is increased, then this perturbation is kept, otherwise a negative unit perturbation ($-\Delta h$) is applied to this groove. If the new FOM is calculated to increase, then this negative perturbation is kept, otherwise it proceeds to the next groove. The guessed height distribution is updated accordingly. One iteration stops when all grooves are traversed. Termination conditions guarantee convergence, such as a maximum number of total iterations or a minimum FOM improvement threshold between two iterations.

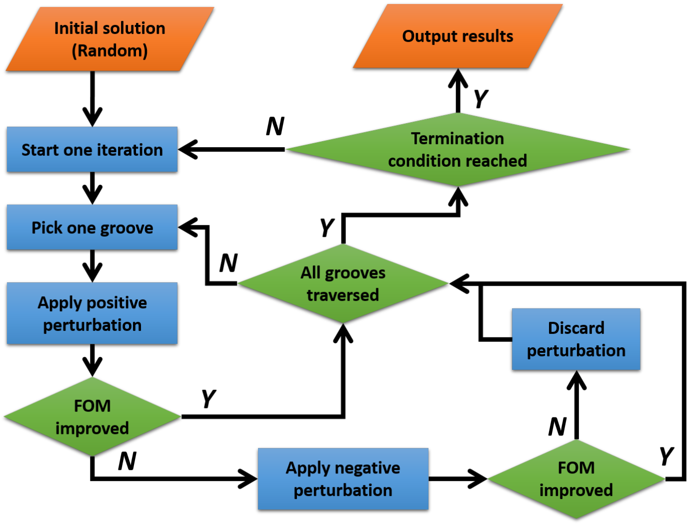

The key design parameters are described below.

Table S1: *Design parameters.*

NA	*f*	*Ring width*	*Max. ring height*	*Number of gray-levels*
0.05	*1mm*	*3μm*	*2.4μm*	*100*
0.18	*1mm*	*1.2μm*	*2.6μm*	*100*

2. Simulation of focusing performance

The simulated point-spread functions (focal spots) at the design wavelengths for the 2 lenses are summarized in Fig. S1. The measured focal spots (from fig. 1 of the main text) are also included for comparison. It can be seen that the measurements agree well with the simulations. These simulations were performed using scalar diffraction theory.

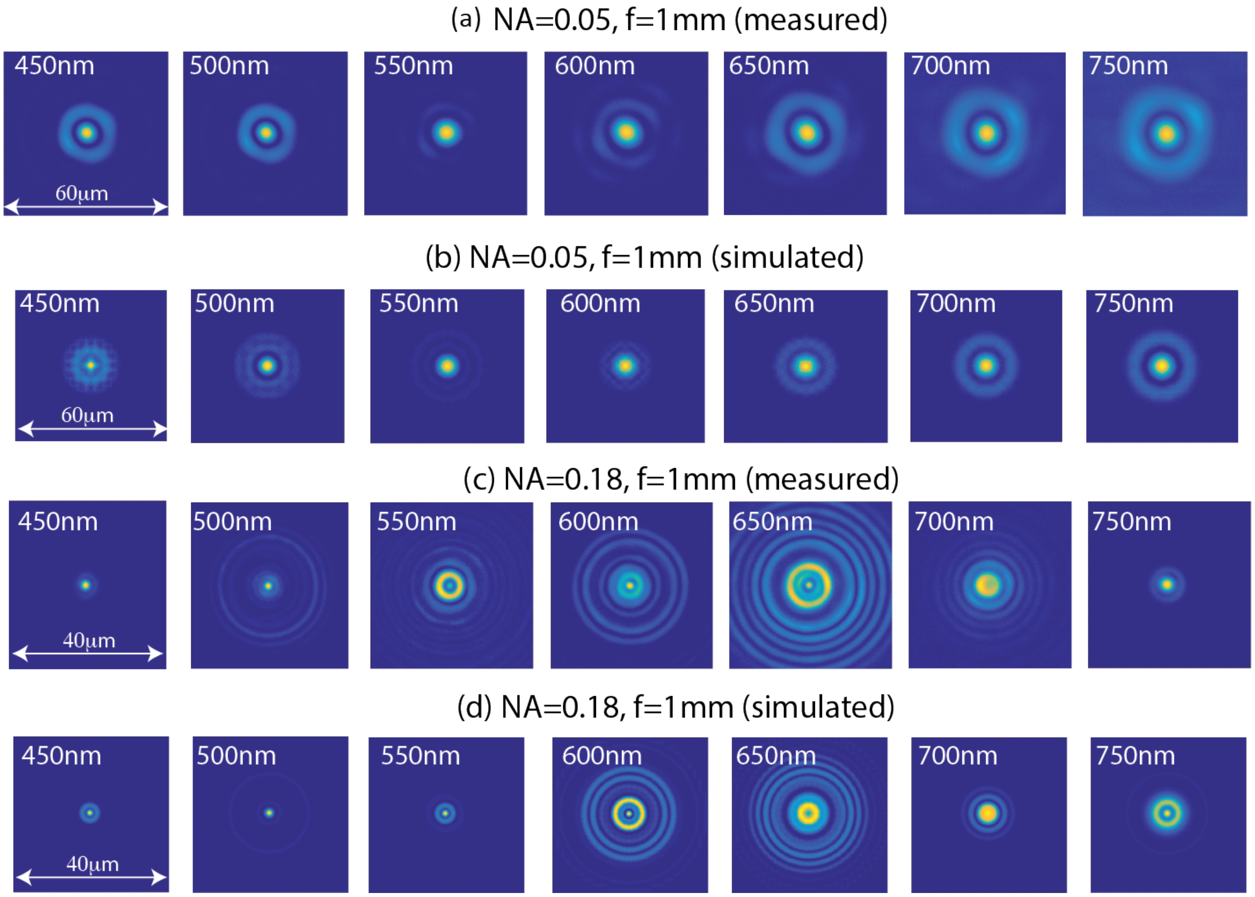

Figure S1: *Focal spots of NA=0.05 lens (a) measured, (b) simulated and those of NA=0.18 lens (c) measured and (d) simulated.*

The simulated focusing efficiency spectra of the 2 designed lenses are shown in Fig. S2. The simulated average efficiency is somewhat higher than the measured ones. The reasons for this is not clear at the moment and we strongly suspect the impact of fabrication errors as described below.

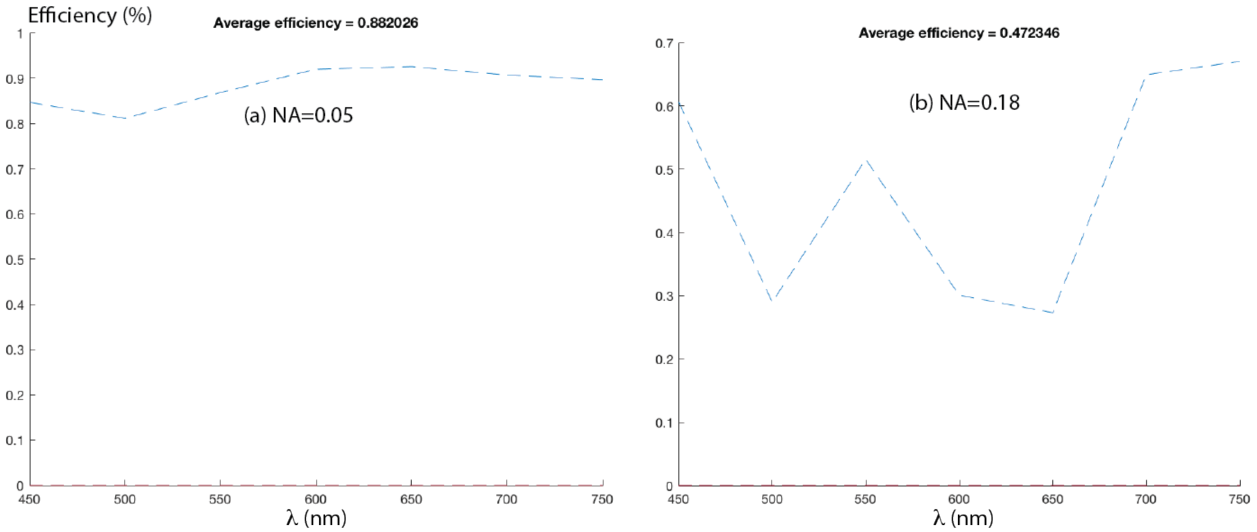

Figure S2: *Simulated focusing efficiency spectrum for (a) NA=0.05 and (b) NA=0.18 lens.*

3. Impact of fabrication errors

We measured the heights of the fabricated design (NA=0.18) over 10 randomly selected rings and these are summarized in the top chart in Fig. S3 along with the corresponding ideal design heights. The estimated error has a mean of 968nm and standard deviation of 156nm. Using this information, we simulated the focusing efficiency for 3 random realizations, where the pixel height error was randomly drawn from an uniform distribution with the same mean and standard deviation as the measured values. The results are summarized in the bottom panel of Fig. S3 and provide more proof for our hypothesis that the fabrication errors are likely reason for the reduced focusing efficiency. Another possibility, which requires additional study is the surface roughness of the photoresist after development.

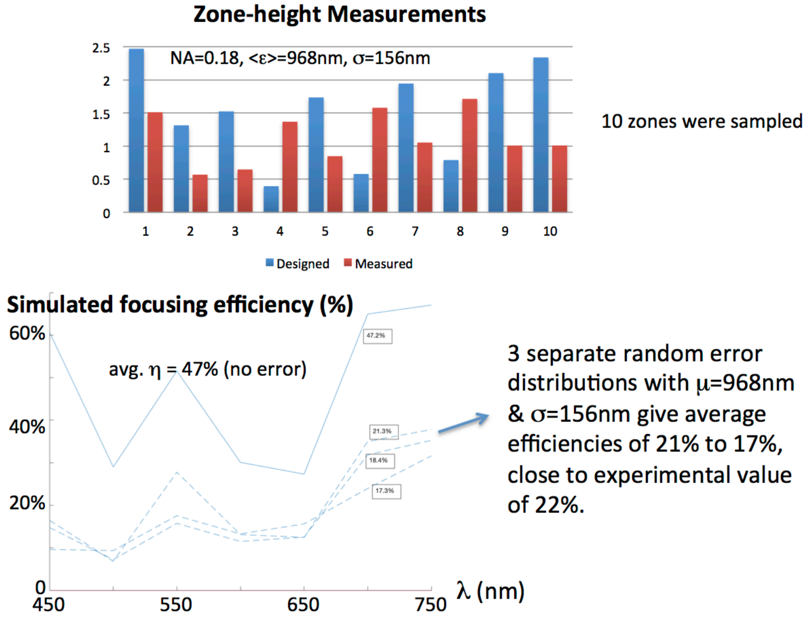

Figure S3: *Top: measured and designed zone heights for 10 randomly selected zones in the NA=0.18 lens. Bottom: Impact of errors in the zone heights on focusing efficiency.*

4. Focal spot characterization setup

The flat lenses were illuminated with expanded and collimated beam from a SuperK EXTREME EXW-6 source (NKT Photonics) and the SuperK VARIA filter (NKT Photonics). The wavelength and bandwidth can be changed using the VARIA filter. The focal planes of the flat lenses were magnified using an objective (RMS20X-PF, Thorlabs) and tube lens (ITL200, Thorlabs) and imaged onto a monochrome sensor (DMM 27UP031-ML, Imaging Source). The setup is shown in Fig. S4. Here, f represents the focal length of the flat lens and w.d. (roughly 2mm) is the working distance of the objective. The gap between objective and tube lens was ~90 mm and that between the sensor and the backside of tube lens was about 148mm. The magnification of the objective-tube lens was 22.22X.

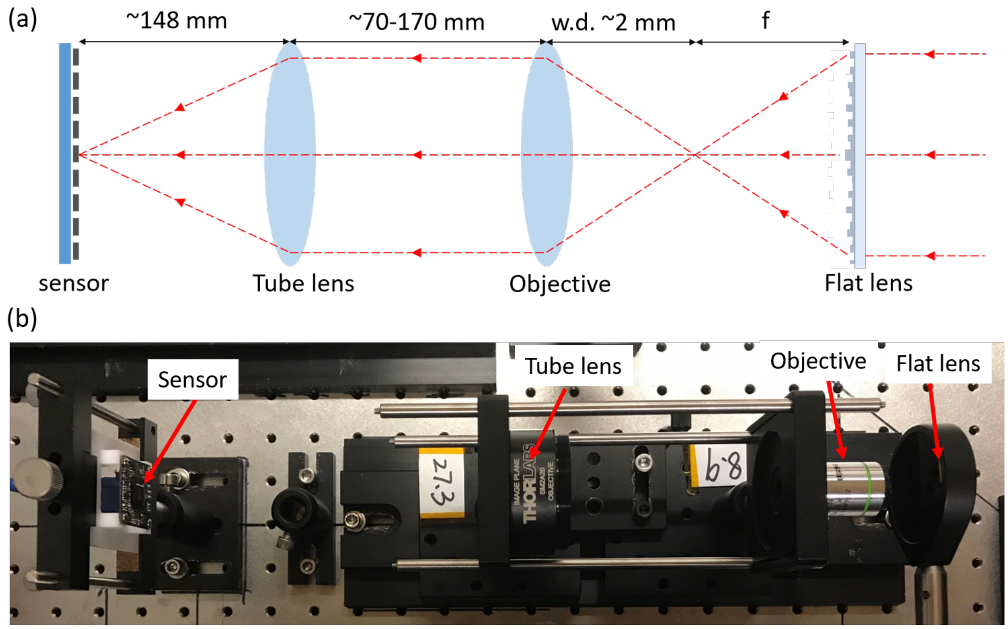

Fig. S4: *(a) Schematic of the setup for focal spot characterization. (b) Photograph of the setup.*

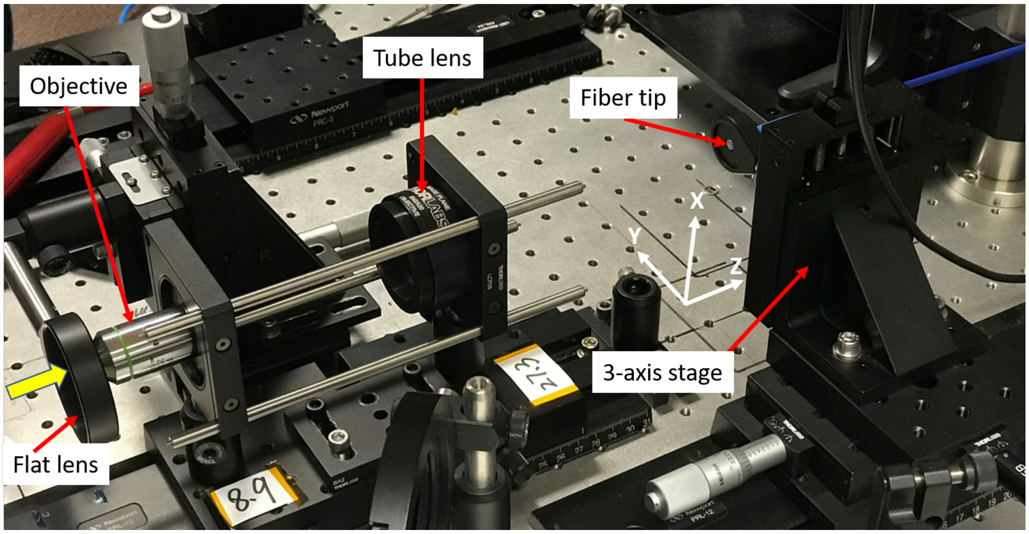

Fig. S5: *Schematic of the setup for focusing efficiency measurement.*

To experimentally determine the focusing efficiency, we used the same setup but now we replaced the monochrome sensor with a 400 µm core diameter fiber tip (P400-1-UV-VIS, Ocean Optics) which in turns was connected to a spectrometer (Jaz Spectrometer, Ocean Optics). The setup is shown in Fig. S5. The flat lens was illuminated with expanded and collimated beam from the SuperK (445nm to 755nm). The fiber tip was scanned in X and Y directions using motorized stages so that the fiber tip was aligned with the peak of the magnified focal spot of the flat lens. A slight adjustment in the Z direction was also made to ensure that the integrated signal on the spectroscope was maximum. A reference signal was recorded with light passing through the unpatterned photoresist. Focusing efficiency was then calculated using the following equation: Focusing efficiency = (spectrometer signal when fiber tip aligned to the peak of magnified psf) / [(reference signal) X (area of magnified lens aperture) / (area of fiber tip aperture)]

5. Imaging setup

For imaging experiment, the 1951 USAF resolution test chart (R3L3S1N, Thorlabs) was used as the object. The flat lenses were used for imaging the object on to the sensor. A diffuser was placed behind the USAF target. The experimental setup is shown in Fig. S6. The USAF target was illuminated with the design wavelengths with 10nm bandwidth and corresponding images were captured using a monochrome sensor (DMM 27UP031-ML, Imaging Source). The exposure time was adjusted to ensure that the images did not get saturated. In each case, a dark frame was recorded and subtracted from the USAF target images.

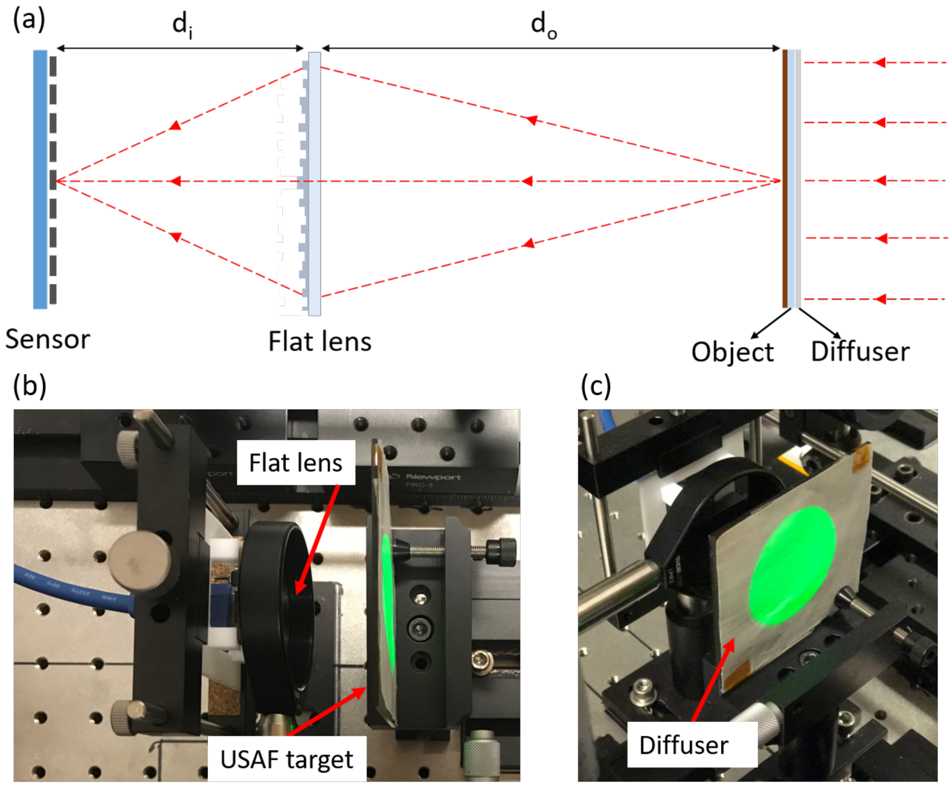

Fig. S6: *(a) Schematic of the imaging setup. (b) and (c) Photographs of the setup.*

Table S2*: Focal length, magnification, object and image distance*

Lens NA	Object distance (mm)	Image distance (mm)	Focal length (mm)	Magnification
0.05	42.3	1.0242	1	0.0242
0.18	38.8	1.0265	1	0.0264

6. Color camera setup

Fig. S7 shows the photographs of the color camera and its components. The color camera consists of a color sensor (DFM 72BUC02-ML), the flat lens and an IR-cut filter. These components were put onto a cage mount which was placed inside a 3D printed enclosure. The IR-cut filter was used only for outdoor photography.

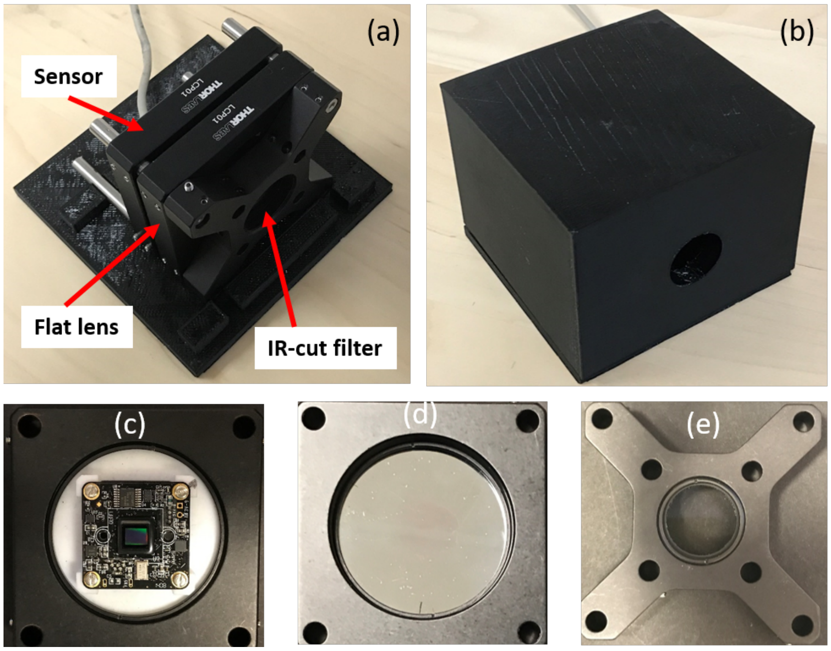

Fig. S7: *(a) Photograph of the color camera. (b) a 3D printed enclosure. Components of the camera: (c) color sensor, (d) flat lens and (e) IR-cut filter.*
